# A Genome-Wide Survey of MATE Transporters in Brassicaceae and Unveiling Their Expression Profiles under Abiotic Stress in Rapeseed

**DOI:** 10.3390/plants9091072

**Published:** 2020-08-20

**Authors:** Cailin Qiao, Jing Yang, Yuanyuan Wan, Sirou Xiang, Mingwei Guan, Hai Du, Zhanglin Tang, Kun Lu, Jiana Li, Cunmin Qu

**Affiliations:** 1 Chongqing Engineering Research Center for Rapeseed, College of Agronomy and Biotechnology, Southwest University, No. 2 Tiansheng Road, Beibei, Chongqing 400715, China; qcl123@email.swu.edu.cn (C.Q.); yj18203807@email.swu.edu.cn (J.Y.); wyywinyuan@email.swu.edu.cn (Y.W.); x231915@email.swu.edu.cn (S.X.); ghhooo1770@email.swu.edu.cn (M.G.); dh20130904@swu.edu.cn (H.D.); tangzhlin@swu.edu.cn (Z.T.); drlukun@swu.edu.cn (K.L.); 2 Academy of Agricultural Sciences, Southwest University, Chongqing 400715, China

**Keywords:** MATE transporters, Brassicaceae, *Brassica napus*, phylogenetic analysis, abiotic stress, expression profiles

## Abstract

The multidrug and toxic compound extrusion (MATE) protein family is important in the export of toxins and other substrates, but detailed information on this family in the Brassicaceae has not yet been reported compared to *Arabidopsis thaliana*. In this study, we identified 57, 124, 81, 85, 130, and 79 *MATE* genes in *A. thaliana*, *Brassica napus*, *Brassica oleracea*, *Brassica rapa*, *Brassica juncea*, and *Brassica nigra*, respectively, which were unevenly distributed on chromosomes owing to both tandem and segmental duplication events. Phylogenetic analysis showed that these genes could be classified into four subgroups, shared high similarity and conservation within each group, and have evolved mainly through purifying selection. Furthermore, numerous *B. napus* *MATE* genes showed differential expression between tissues and developmental stages and between plants treated with heavy metals or hormones and untreated control plants. This differential expression was especially pronounced for the Group 2 and 3 *BnaMATE* genes, indicating that they may play important roles in stress tolerance and hormone induction. Our results provide a valuable foundation for the functional dissection of the different *BnaMATE* homologs in *B. napus* and its parental lines, as well as for the breeding of more stress-tolerant *B. napus* genotypes.

## 1. Introduction

In nature, plants are exposed to various exogenous and endogenous toxins during their lifespans, and they have developed a series of adaptive response mechanisms to dispose of and detoxify toxic compounds [1]. A group of secondary transporters first reported in *Vibrio parahaemolyticus* and *Escherichia coli* [2,3], the MATE (multidrug and toxic compound extrusion or multi-antimicrobial extrusion)/DTX (detoxification efflux carrier) family, is a universal family of proteins involved in the metabolism of toxic compounds and organic acids. Members of the MATE family are composed of 400–700 amino acids and contain 12 transmembrane helices [1,4]. However, the *MATE* gene family lacks a conserved consensus sequence, and MATE proteins share only about 40% sequence similarity [1]. Numerous studies indicate that MATE family proteins play widespread roles in various biological processes in plants, such as secondary metabolite transport [5,6,7,8,9,10], disease resistance [11,12], the detoxification of heavy metals [13,14,15], and the transport of plant hormones [16,17,18]. Numerous studies have characterized *MATE* genes from a wide range of living organisms, showing that they have even greater diversity in plant genomes than in mammalian genomes [19]. For example, 56 *MATE* genes have been isolated from *Arabidopsis thaliana* [13]. The *MATE* gene family has also been widely characterized in other plant species, including rice (*Oryza sativa*) (52) [20,21], maize (*Zea mays*) (49) [22], tomato (*Solanum lycopersicum*) (67) [4], upland cotton (*Gossypium hirsutum*) (72) [23], and soybean (*Glycine max*) (117) [24]. Recently, 67 MATE genes were identified in the *C. sinensis* genome [25]. However, genome-wide analysis of the *MATE* family in Brassicaceae species has been limited, and no such studies have been reported in rapeseed (*Brassica napus*).

In the Brassicaceae, the relationships of three diploid species (*B. rapa*, AA, 2*n* = 20; *B. nigra*, BB, 2*n* =  16; and *B. oleracea*, CC, 2*n* = 18) and three amphidiploid plants (*B. juncea*; AABB, 2*n* = 36; *B. napus*, AACC, 2*n* =  38; and *Brassica carinata*, BBCC, 2*n* = 34) have been thoroughly described according to the “triangle of U” theory [26,27], which also provides an excellent evolutionary model to investigate the expansion of gene families [28,29]. During the course of evolution, the Brassicaceae have experienced ɑ and β duplication events, as well as a triplication event specific to the *Brassica* clade compared with the model plant *A. thaliana* [30]. These whole-genome duplication (WGD) events, along with the merger of the two progenitor genomes, have resulted in copious gene duplication in the *B. napus* genome, followed by substantial gene loss [31]. Thus, Brassica is regarded as an ideal model for investigating the evolution of polyploidy [29]. However, no systematic and comprehensive study of this family is reported among Brassicaceae species. To date, with the completion of five Brassicaceae species (*B. napus*, *B. oleracea*, *B. rapa*, *B. juncea*, and *B. nigra*) genome sequencing [29,32,33,34,35,36], an investigation of the *MATE* gene family in Brassicaceae is now feasible and can not only provide insights into the evolutionary mechanisms of hybridization (allopolyploidy) among these species, but also lay a theoretical basis for a clearer understanding of the mechanisms involved in plant stress defense.

In present study, we identified *MATE* family genes in the Brassicaceae genomes available in the Brassica database (BRAD) [37] using the 57 MATE sequences from *A. thaliana* as queries. Then, we performed a detailed phylogenetic analysis of the structure, motifs, chromosome distribution, and duplication of these genes, as well as an evolutionary analysis. We also investigated the expression profiles of *MATE* genes in different tissues of *B. napus* and during responses to hormone treatment and heavy metal stress. Our results provide important information about the origin and evolution of the *MATE* family in Brassicaceae and lay a foundation for the further functional characterization of *MATE* genes in *B. napus*.

## 2. Results

### 2.1. Identification of MATE Transporters in Brassicaceae Species

**Figure 1:**
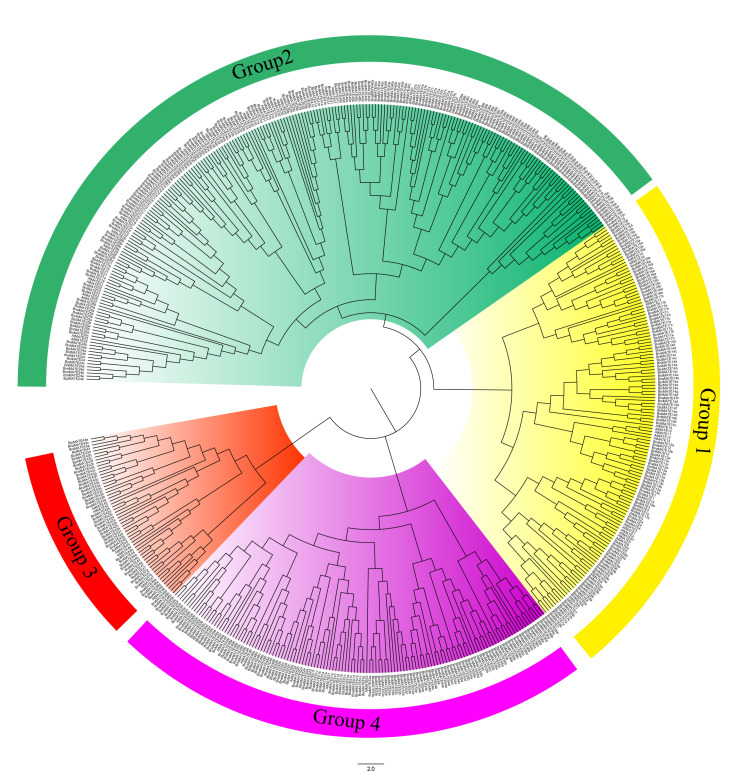
Phylogenetic analysis of the multidrug and toxic compound extrusion (MATE) transporters. The rooted neighbor-joining phylogenetic tree was constructed using MEGA7.0 and visualized using Figure Tree v1.4.2. There are 556 MATEs in *A. thaliana* and five Brassicaceae species, including 57 AtMATEs, 124 BnaMATEs, 85 BraMATEs, 81 BolMATEs, 79 BniMATEs, and 130 BjuMATEs. These MATE transporters are divided into four subgroups, Groups 1–4, which are marked in yellow, green, red, and purple, respectively. The outer circle represents the four groups.

Using 57 AtMATE protein sequences as queries, we identified 499 putative *MATE* genes from various Brassicaceae species, including 124 *BnaMATE*, 85 *BraMATE*, 81 *BolMATE*, 79 *BniMATE*, and 130 *BjuMATE* genes. Based on their homology relationship with the respective *A. thaliana* genes, these genes were named *BnaMATE1* to *BnaMATE57* for *B. napus*, *BraMATE1* to *BraMATE57* for *B. rapa*, *BolMATE1* to *BolMATE57* for *B. oleracea*, *BniMATE1* to *BniMATE57* for *B. nigra* and *BjuMATE1* to *BjuMATE57* for *B. juncea*, respectively (Figure 1, Table S2). Eight *AtMATE* genes (*AtMATE2*, *AtMATE10*, *AtMATE11*, *AtMATE20*, *AtMATE22*, *AtMATE26*, *AtMATE32,* and *AtMATE57*) had no orthologs among the other five species, which was indicative of gene loss during evolution between *A. thaliana* and *Brassica* species.

Basic information about these *MATE* genes in Brassicaceae species, including name, locus, molecular weight, subcellular localization, and other related information, is listed in Table S2. In *B. napus*, we found that *BnaMATE* genes encode proteins of 124 (*BnaMATE56d*) to 971 (*BnaMATE15c*) amino acids, with an average length of 489 amino acids. The relative molecular weights (MWs) ranged from 13.07 kDa (*BnaMATE56d*) to 107.53 kDa (*BnaMATE15c*), and theoretical pI (isoelectric point) values ranged from 4.89 (*BnaMATE40d*) to 10.55 (*BnaMATE17d*). Similarly, individual MATE proteins varied in length and physicochemical properties among the other five species (Table S2). These results suggest that MATE transporters might have changed between species to serve different functions.

Previous studies have localized MATE transporters to the plasma membrane, chloroplast, cytoplasm, vacuole, and endoplasmic reticulum, as well as extracellularly [24,38,39]. Here, we found the highest proportion localized in the plasma membrane (109 genes), accounting for >80% of all *BnaMATE* genes in *B. napus*; four genes were expressed in chloroplasts and 11 genes were expressed in the vacuoles. The trend in the other four species was similar, with 91.8% (*B. rapa*), 81.5% (*B. oleracea*), 91.5% (*B. juncea*), and 83.5% (*B. nigra*) of *MATE* genes, respectively (Table S2), expressed in the plasma membrane, which was consistent with previous results [24,39]. This strong representation of MATEs in the plasma membrane is consistent with their primary role of maintaining membrane integrity through the exclusion of toxins from plants.

### 2.2. Phylogenetic Analysis of the MATE Transporters

To investigate the evolutionary relationships among MATE transporters between *A. thaliana* and various Brassicaceae species, we divided the 556 MATE transporters into four categories, denoted Groups 1–4 (Table 1 and Figure 1). Group 2 was the largest clade, with 230 members (24 in *A. thaliana*, 51 in *B. napus*, 37 in *B. rapa*, 34 in *B. oleracea*, 44 in *B. juncea,* and 40 in *B. nigra*), and Group 3 was the smallest, with 59 members (6 in *A. thaliana*, 15 in *B. napus*, 10 in *B. rapa*, 7 in *B. oleracea*, 15 in *B. juncea,* and 6 in *B. nigra*); Groups 1 and 4 contained 137 and 130 members, respectively (Figure 1). All of the MATE transporters we identified in Brassicaceae were classified into previously defined subgroups along with *A. thaliana* MATE proteins, suggesting that they might share similar functions within the subfamilies. In addition, the classifications were consistent with those of soybean MATE transporters [1,23,24], indicating that the classification adopted in this study was accurate and conforms to previous findings about plant MATE proteins.

### 2.3. Chromosomal Location of MATE Genes in Brassicaceae Genomes

**Figure 2:**
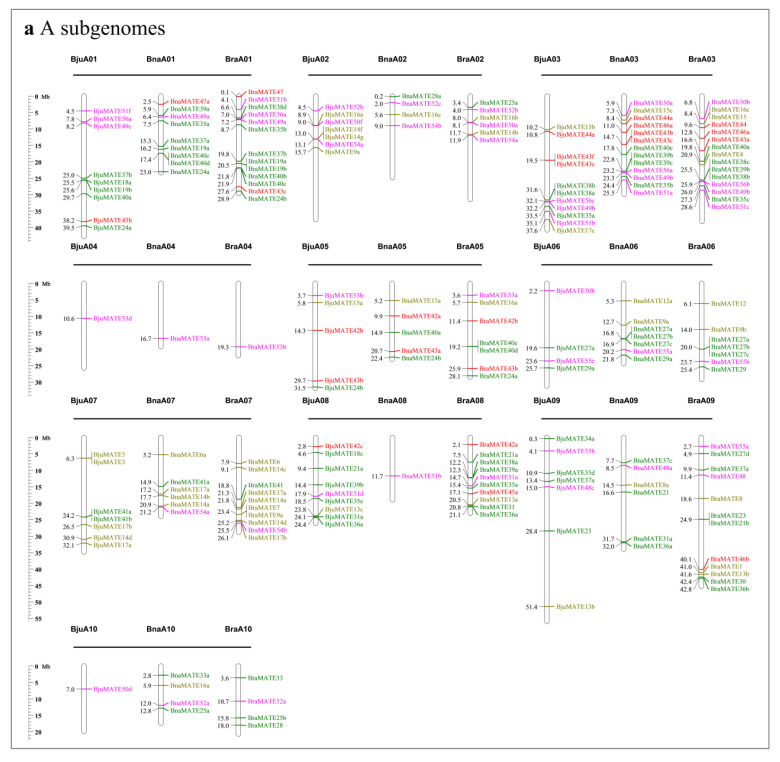

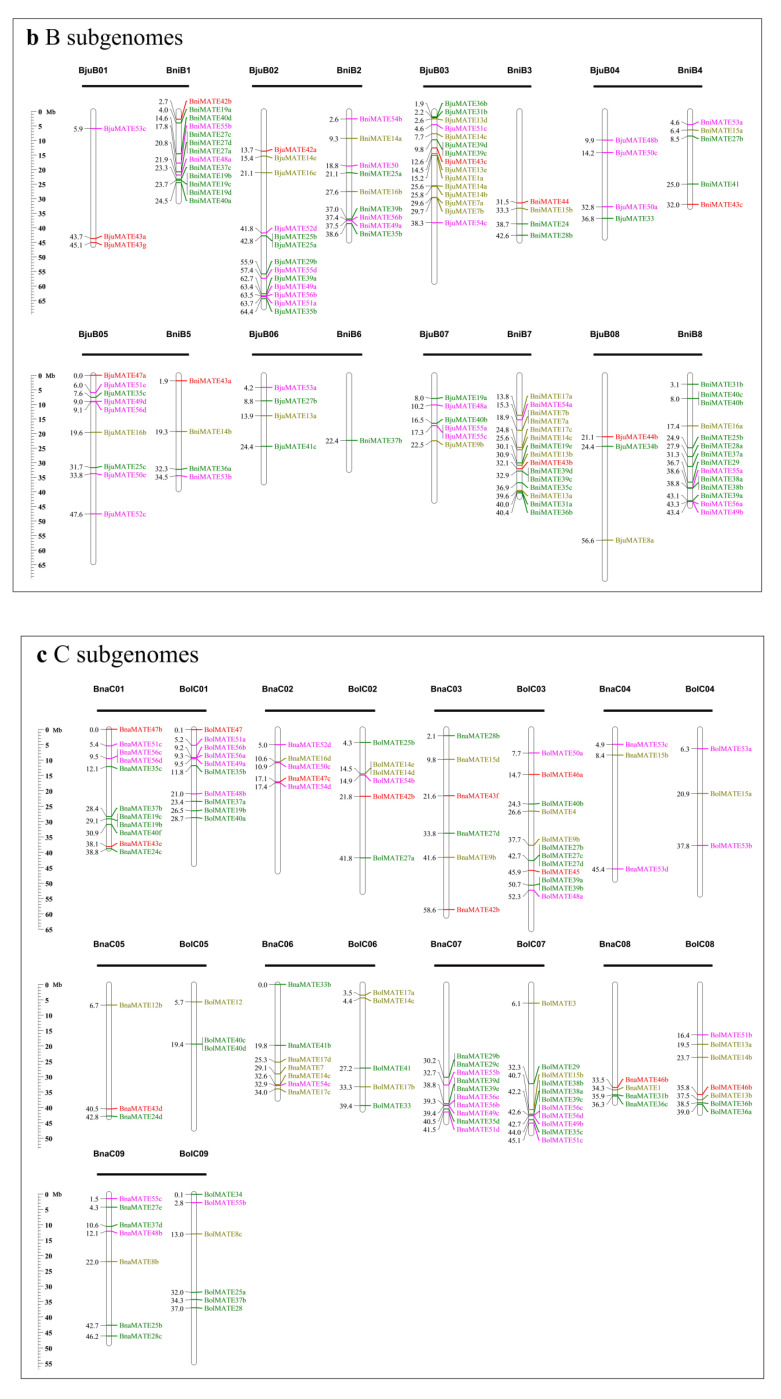
Chromosomal distribution and analysis of duplication events in *MATE* family genes among Brassicaceae species. (**a**) Chromosomal distribution and analysis of duplication events in *MATE* family genes among A subgenome. (**b**) Chromosomal distribution and analysis of duplication events in *MATE* family genes among B subgenome. (**c**) Chromosomal distribution and analysis of duplication events in *MATE* family genes among C subgenome. Genes from the same subgroups are indicated by the same color, which is consistent with that for the corresponding family in the phylogenetic tree (Figure 1); genes located on the scaffold are not shown. The labels on the corresponding chromosomes indicate the source organism and the subgenome. The scales indicate the sizes of the various *Brassica* genomes (Mb). Bra, *B. rapa*; Bol, *B. oleracea*; Bni, *B. nigra*; Bna, *B. napus*; Bju, *B. juncea*.

Based on the physical positions of these *MATE* genes in the Brassicaceae genomes, 443 of the 499 full-length *MATE* genes were accurately mapped onto 64 chromosomes among the five Brassicaceae species (202 in the A_n_ genome, 122 in the B_n_ genome, and 119 in the C_n_ genome) (Figure 2), whereas 56 *MATE* genes were assigned to random chromosomes (12), scaffold chromosomes (34), and contig chromosomes (10). Among them, 56 and 56 *BnaMATE* genes were distributed on the A_na_ and C_na_ subgenomes of *B. napus*; 61 and 57 *BjuMATE* genes were mapped to the A_ju_ and B_ju_ subgenomes of *B. juncea*; and 85 *BraMATE* genes were located on the A_ra_ subgenomes of *B. rapa*, 65 *BniMATE* genes were located on the B_ni_ subgenomes of *B. nigra*, and 63 *BolMATE* genes were located on the C_ol_ subgenomes of *B. oleracea* (Figure 2). However, the sum of the *MATE* genes from *B. rapa* (AA) and *B. oleracea* (CC) or from *B. rapa* (AA) and *B. nigra* (BB) in each subgenome was larger than that from *B. napus* (AACC) and *B. juncea* (AABB), suggesting that the *MATE* genes underwent a notable gene loss event during the evolution of *B. napus*, *B. juncea,* and their relatives. In addition, these genes were unevenly distributed on the chromosomes. For instance, regions dense in *MATE* genes were discovered on chromosomes A03, C01, and C07 in B. napus (Figure 2). In *B. oleracea*, chromosome C03 contained the greatest number of *MATE* genes, 12, whereas C04 and C05 contained three *MATE* genes. In *B. rapa*, chromosome A03 contained the most *MATE* genes, 15, whereas A04 contained two *MATE* genes. In *B. nigra*, chromosome B7 contained the most *MATE* genes, 17, and B6 contained one. In *B. juncea*, chromosome B03 contained the most *MATE* genes, 15, whereas A04 and A10 had only one (Figure 2), suggesting that they were unevenly distributed on each chromosome of Brassicaceae species.

### 2.4. Synteny and Gene Duplication Analysis of MATE Transporters

**Figure 3:**
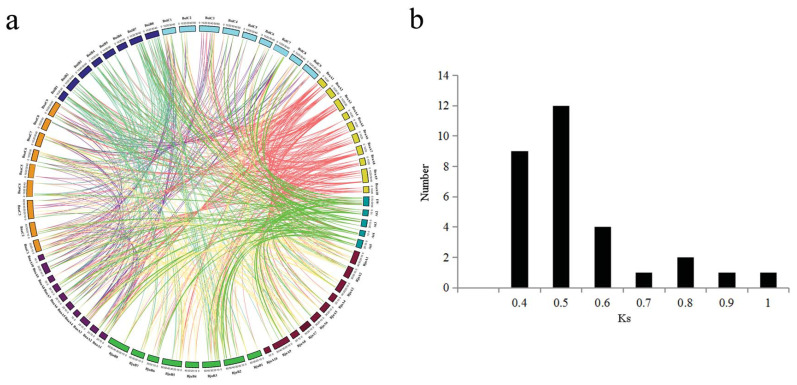

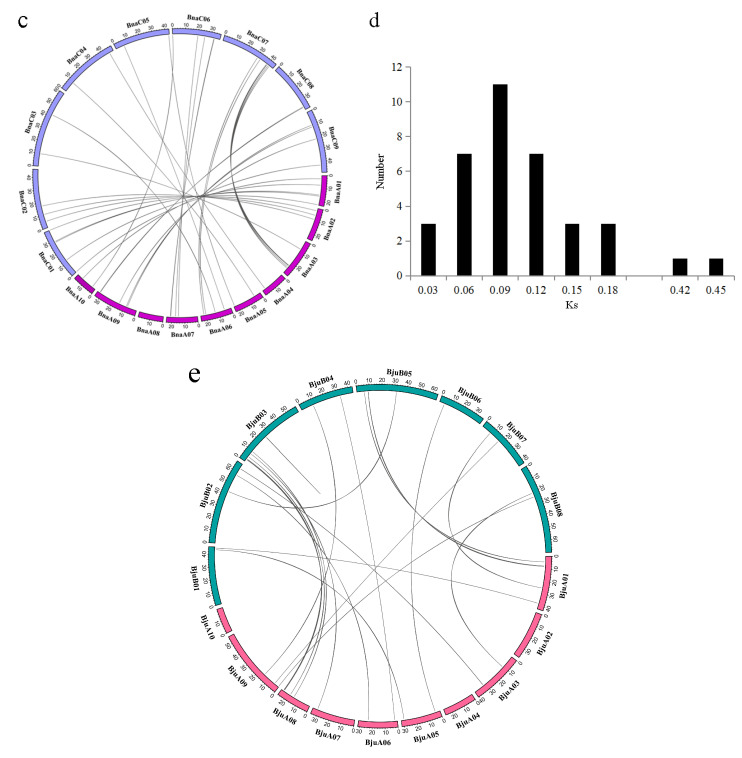
Duplicated and orthologous genes in the Brassicaceae. (**a**) Circle plot of orthologous genes in *A. thaliana* (At), *B. oleracea* (Bol), *B. rapa* (Bra), *B. napus* (Bna), *B. juncea* (Bju), and *B. nigra* (Bni). (**b**) Density of K_s_ values of *MATE* gene pairs between *B. napus* and *A. thaliana*. (**c**) Duplicate gene pairs in *B. napus*. (**d**) Density of K_s_ values of duplicated *MATE* genes in *B. napus*. (**e**) Duplicate gene pairs in *B. juncea*.

To explore the entire evolutionary history of each *MATE* genes, we performed comparative analysis of *MATE* genes between *A. thaliana* and five Brassicaceae genomes (Figure 3a) and determined their patterns of orthologous gene retention or loss based on their collinearity relationships. In total, 40 *A. thaliana MATE* genes showed collinear relationships with the five Brassicaceae species. Among them, we identified 30 collinear gene pairs between *A. thaliana* and *B. napus*, 17 collinear gene pairs between *A. thaliana* and *B. oleracea*, 29 collinear gene pairs between *A. thaliana* and *B. rapa*, 23 collinear gene pairs between *A. thaliana* and *B. juncea,* and 15 collinear gene pairs between *A. thaliana* and *B. nigra* (Table S3). In addition, 52 gene pairs maintained their relative positions between the A_na_ subgenome of *B. napus* and the A_ra_ genome of *B. rapa*, and 44 gene pairs maintained their relative positions between the C_na_ subgenome of *B. napus* and the C_ol_ genome of *B. oleracea*. Furthermore, 29 gene pairs maintained their relative positions between the A_ju_ subgenome of *B. juncea* and the A_na_ genome of *B. napus*, and 42 gene pairs maintained their relative positions between the B_ju_ subgenome of *B. juncea* and the B_ni_ genome of *B. nigra*.

Of the 101,040 genes in the *B. napus* genome, 997 genes (0.99%) appeared to have undergone tandem duplication and 74,183 genes (73.4%) appeared to have undergone WGD or segmental duplication (Table S4), which showed less tandem duplication and more WGD or segmental duplication compared with the results of a previous study [40]. Furthermore, 113 out of 124 *BnaMATE* genes (91.1%) were derived from WGD or segmental duplication, which was a number slightly larger than the average percentage at the whole-genome level (73.4%). Therefore, it appears that WGD or segmental duplication played an important role in the expansion of the MATE family in *B. napus*. Likewise, we also examined the *MATE* gene expansion patterns in *B. rapa*, *B. oleracea*, *B. nigra,* and *B. juncea*, and we showed that all of the *BraMATE* genes (100%; 85/85) and most of the *BolMATE* (95.1%; 77/81), *BniMATE* (93.7%; 74/79), and *BjuMATE* genes (90.8%; 118/130) were derived from segmental duplication, followed by dispersed duplication (0% in *B. rapa*, 3.7% in *B. oleracea*, 3.8% in *B. nigra,* and 7.7% in *B. juncea*) (Table S4).

In addition, we used the synonymous base substitution rates (*K*_s_ values) as a proxy for time to estimate the approximate ages of the segmental duplication events [1]. Then, we predicted the *K*_s_ values for all collinear gene pairs. These ranged from 0.3322 to 0.9765 between *B. napus* and *A. thaliana*, with a mean of 0.4903 (Figure 3b). The divergence time ranged from 11.07 to 32.55 million years ago (MYA), with a mean of 16.34 MYA, indicating that the *MATE* genes of *B. napus* and *A. thaliana* diverged approximately 16 MYA. This is consistent with the likely timing of the recent whole-genome triplication event in *Brassica*, which has been estimated at approximately 9−15 MYA or even 28 MYA [41]. In addition, we identified 36 and 26 duplicate gene pairs in *B. napus* and *B. juncea*, respectively (Figure 3c,e). Then, we estimated the timing of the WGD event based on the distribution of *K*_s_ values, which ranged from 0.0192 to 0.4286 (mean, 0.135) in *B. napus* duplicate gene pairs and from 0.0310 to 0.2591 (mean, 0.1684) in *B. juncea* duplicate gene pairs. In *B. napus*, the corresponding duplication time range was 0.64−14.29 MYA (mean, 3.45 MYA), whereas in *B. juncea*, it was 1.03−8.64 MYA (mean, 5.61 MYA) (Table S5). One peak (0.06–0.09) in *K*_s_ values was observed in *B. napus*, representing the duplication time of these genes, which occurred during the formation of *B. napus* 7500–12,500 years ago (Figure 3d). Therefore, the processes of Brassicaceae speciation and whole-genome triplication likely played an important role in the divergence of the duplicated *MATE* genes among these species.

We also calculated the rates of nonsynonymous substitutions (*K*_a_) and synonymous substitutions (*K*_s_) and the *K*_a_/*K*_s_ ratios of the *MATE* gene pairs to identify the evolutionary constraints acting on the *MATE* gene pairs, and we found that the *K*_a_/*K*_s_ ratios of the duplicates were commonly less than 1 (Table S5), indicating that these genes were subject to purifying selection.

### 2.5. Analysis of MATE Gene Structures and Conserved Motifs between A. thaliana and B. napus

**Figure 4:**
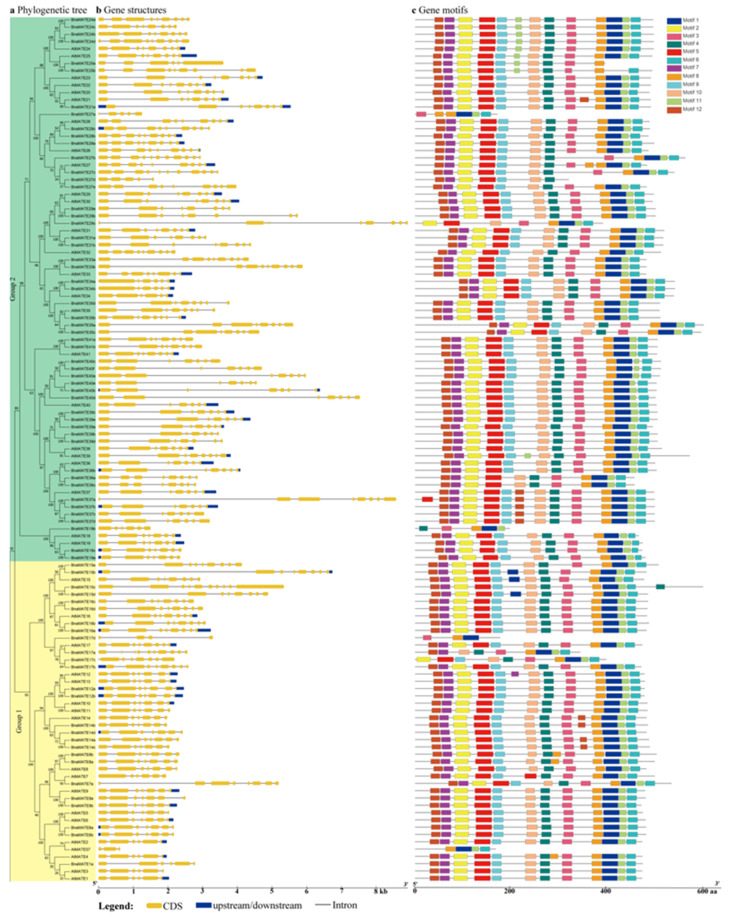

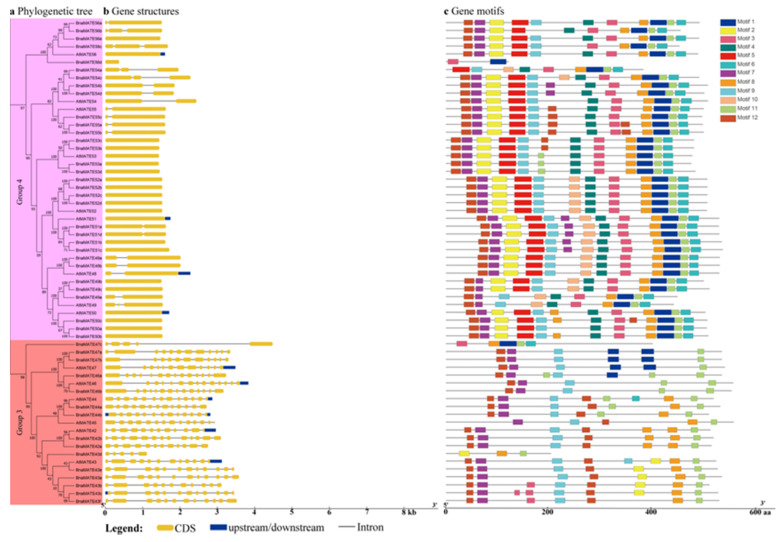
Gene structure and conserved motifs in *MATE* genes from *Brassica napus* and *Arabidopsis thaliana.* (**a**) The neighbor-joining tree was generated from the alignment of 181 *MATE* genes from *A. thaliana* and *B. napus*. The different background colors represent the four subgroups. (**b**) Gene structures were generated using the Gene Structure Display Server (GSDS 2.0) [42]. Yellow boxes indicate exons, black lines indicate introns, and blue boxes indicate untranslated regions (UTRs) of genes. (**c**) Conserved motifs were detected using MEME and are shown as boxes of different colors (numbered as motifs 1–12).

The allotetraploid Brassicaceae species rapeseed (*B. napus*) is one of the main oilseed crops worldwide, serving as a source of edible oil, biodiesel, and protein-rich animal feed. To provide a clear functional characterization of the *BnaMATE* genes, as well as to enable the manipulation of traits related to rapeseed metabolism, we analyzed the intron–exon structures of these genes using GSDS v2.0 (http://gsds.cbi.pku.edu.cn/index.php); the results are displayed along with the subgroups of phylogenetic tree (Figure 4a). Notably, the lengths and numbers of exons and introns were more similar within the same subgroup (Figure 4b). For example, the number of exons ranged from 5 to 11 in the largest group, Group 2, and the maximum number of exons (11–14 exons) was found in Group 3, except for *BnaMATE43d* and *BnaMATE47c*, which have only 5 exons. The genes in Group 4 generally had the smallest number of exons, 1–3, and the *MATE* genes in Group 1 had 4–10 exons. Our findings were in agreement with previous studies reporting that *MATE* genes from different groups were generally distinct, with each group sharing a common gene structural layout [22]. In addition, similar genes structures were observed among *B. rapa*, *B. oleracea*, *B. nigra,* and *B. juncea*, which were divided into the same subfamilies (Figure 1, Table S2).

Next, we identified the conserved motifs of MATE sequences using the MEME online software (Figure 4c, Figure S1). In most cases, the protein architecture was remarkably conserved within specific subgroups, providing further support to the phylogenetic analysis based on MATE domains (Figure S2) and suggesting that there are functional similarities within the groups. In addition, the types and sequences of the motifs are similar among Group 1, 2, and 4, which contain all 12 conserved motifs, whereas the most distinctive pattern of motifs was found in Group 3 proteins, which contain a smaller number of motifs. These findings suggest that these conserved motifs may have crucial roles in subfamily-specific functions.

### 2.6. Expression Profiles of BnaMATE Genes in Various Rapeseed Tissues

**Figure 5:**
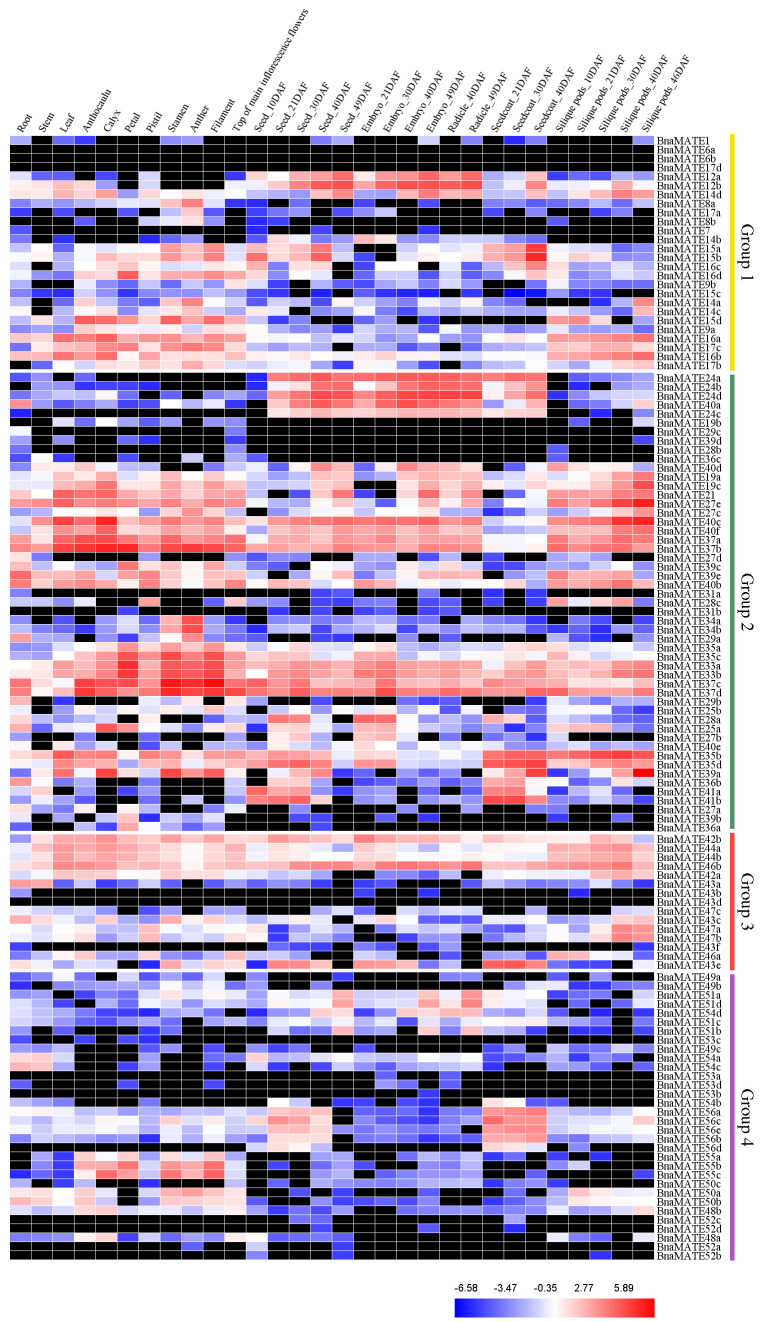
Expression patterns of all 124 *BnaMATE* genes in 15 different tissues at different developmental stages in *B. napus*. The color bar represents log_2_ expression levels (fragments per kilobase per million mapped fragments, or FPKM). Red, blue, and white indicate high, low, and medium expression levels, respectively, and black indicates no expression (FPKM = 0). The abbreviations above the heatmap indicate the different tissues and organs/developmental stages of *B. napus* ZS11 (listed in Table S1).

Based on the transcriptome sequencing datasets from *B. napus* ZS11 (BioProject ID PRJNA358784), we characterized the relative transcript abundances of *BnaMATE* gene transcripts in 15 different tissues of rapeseed (Figure 5, Table S6). Within Group 1, most *BnaMATE* genes showed low or no expression in the tested tissues and organs, such as *BnaMATE8a*, *BnaMATE8b*, *BnaMATE9a*, *BnaMATE9b*, *BnaMATE14a*, *BnaMATE14b*, *BnaMATE14c*, and *BnaMATE17a,* which were expressed at low levels; in contrast, *BnaMATE1*, *BnaMATE6a*, *BnaMATE6b*, *BnaMATE7*, *BnaMATE15c*, and *BnaMATE17d* were barely expressed in the tested tissues and organs. However, *BnaMATEs* also displayed the specifically expression patterns; for example, *BnaMATE12a* and *BnaMATE12b* were more highly expressed in seeds and embryos than in other tissues; *BnaMATE15a* and *BnaMATE15b* were more highly expressed in seeds (40 DAF) and seed coats (40 DAF) than in other tissues; *BnaMATE16a* and *BnaMATE16b* were more highly expressed in the tissues of the whole flower, leaves, and silique pods compared with other tissues; and *BnaMATE17b* and *BnaMATE17c* were more highly expressed in flowers and seed pods than in other tissues (Figure 5).

In Group 2, *BnaMATE19a* and *BnaMATE19c* were more highly expressed in the tissues of flowers and late developmental tissues (at 40 and 49 DAF), including seeds, embryos, and silique pods, than in other tissues; *BnaMATE24a*, *BnaMATE24b*, *BnaMATE24c*, and *BnaMATE24d* were specifically expressed in seeds, embryos, and seed coat; *BnaMATE35a*, *BnaMATE35b*, *BnaMATE35c*, and *BnaMATE35d* were specifically expressed in flower, seed coat, and silique pod tissues; and *BnaMATE40a*, *BnaMATE40c,* and *BnaMATE40f* were highly expressed in seeds and embryos and (for the latter two) in leaves, anthocaulus, calyx, and silique pods. *BnaMATE33a*, *BnaMATE33b*, *BnaMATE37a*, *BnaMATE37b*, *BnaMATE37c,* and *BnaMATE37d* were expressed in all tissues at different developmental stages, and they were more highly expressed in flower tissues (anthocaulus, calyx, petal, pistils, stamens, anthers, filaments, and the tops of main inflorescence flowers). Finally, *BnaMATE19b*, *BnaMATE31a*, *BnaMATE31b*, *BnaMATE28b*, *BnaMATE36c,* and *BnaMATE39d* were barely expressed in any tissue (Figure 5).

Among Group 3 genes, most were expressed in at least one tissue, with the exception of *BnaMATE43b*, *BnaMATE43d*, and *BnaMATE43f*, which were barely detected in any tissue or organ. *BnaMATE44a*, *BnaMATE44b*, and *BnaMATE46b* were highly expressed throughout plant development, whereas *BnaMATE43e* was highly expressed in the seed coat as compared with other tissues (Figure 5).

In Group 4, *BnaMATE55a*, *BnaMATE55b,* and *BnaMATE55c* were specifically expressed in flower tissues (anthocaulus, calyx, petal, pistils, stamens, anthers, filaments, and the tops of main inflorescence flowers), and *BnaMATE56a*, *BnaMATE56b*, *BnaMATE56c*, *BnaMATE56d,* and *BnaMATE56e* were specifically expressed in seeds and seed coat (at 21, 30, and 40 DAF). In addition, the four *BnaMATE52* genes and four *BnaMATE53* genes were barely expressed in any tissues (Figure 5).

Overall, 11 *BnaMATE* genes were expressed in all tissues at different developmental stages, whereas 29 exhibited almost no expression, and 84 were specifically expressed in particular tissues, suggesting that they may play multiple or tissue-specific roles in the tested tissues and organs of *B. napus*.

### 2.7. Expression Patterns of BnaMATE Genes under Heavy Metal Stress

**Figure 6:**
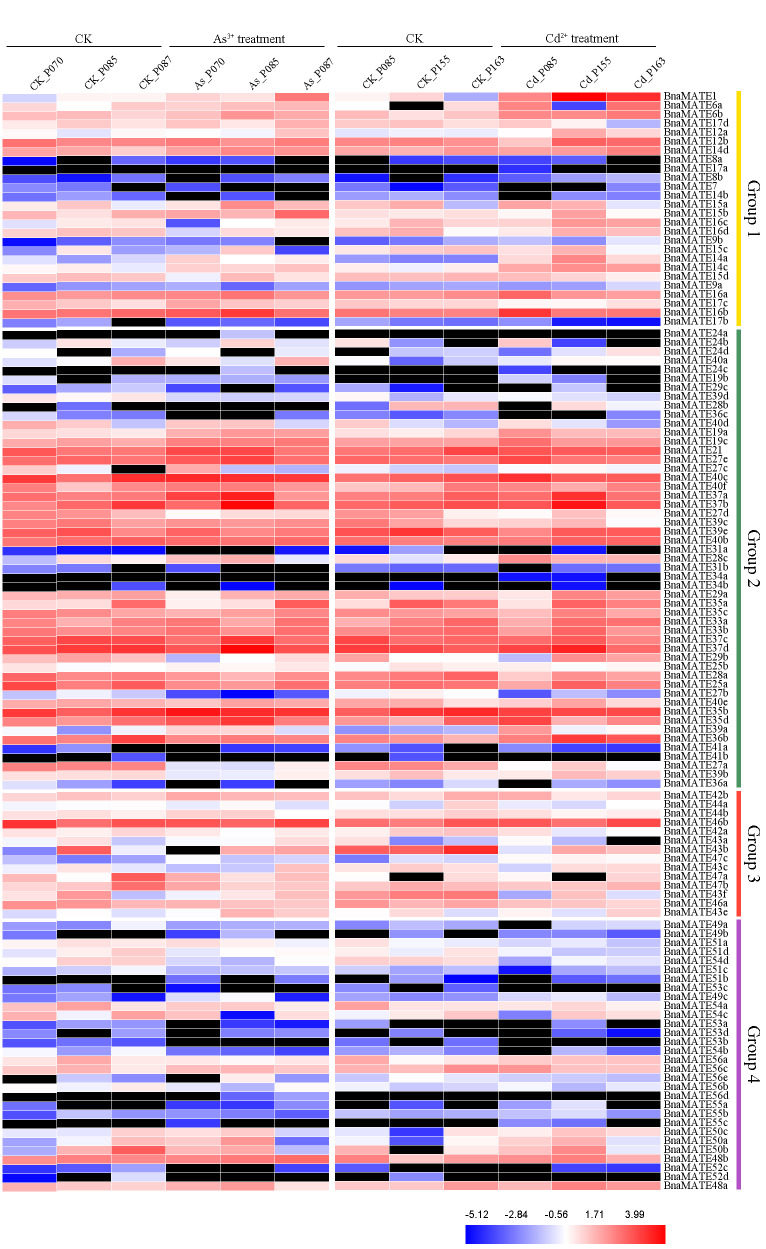
Expression analysis of *BnaMATE* family genes in different rapeseed tissues under As^3+^ and Cd^2+^ treatment. The color bar represents log_2_ expression levels (FPKM). Red, blue, and white indicate high, low, and medium expression levels, respectively, and black indicates no expression (FPKM = 0). The abbreviations above the heatmap indicate the different cultivars of *B. napus* tested (listed in Table S7).

Previous studies showed that the *MATE* genes have important roles in resistance to various abiotic stresses [13,14,15,16,17,18]. Thus, we investigated the expression patterns of the *BnaMATE* genes under As^3+^ and Cd^2+^ stress using RNA-seq. Under normal conditions, the expression patterns of *BnaMATEs* were similar to those identified in the absence of metal ion stress, with different expression profiles detected among different rapeseed varieties (Figure 6, Table S7). For instance, *BnaMATE33a*, *BnaMATE33b*, *BnaMATE37a*, *BnaMATE37b*, *BnaMATE37c*, and *BnaMATE37d* were highly expressed in all tissues, whereas *BnaMATE31a*, *BnaMATE31b*, and the members of *BnaMATE52* and *BnaMATE53* were expressed minimally or repressed. In general, the expression of *BnaMATE* genes in Groups 1, 2, and 4 was clearly induced by As^3+^ or Cd^2+^ treatment, whereas that of Group 3 genes was suppressed, compared with their expression under normal conditions (Figure 6).

### 2.8. Expression Patterns of BnaMATE under Hormone Treatments

**Figure 7:**
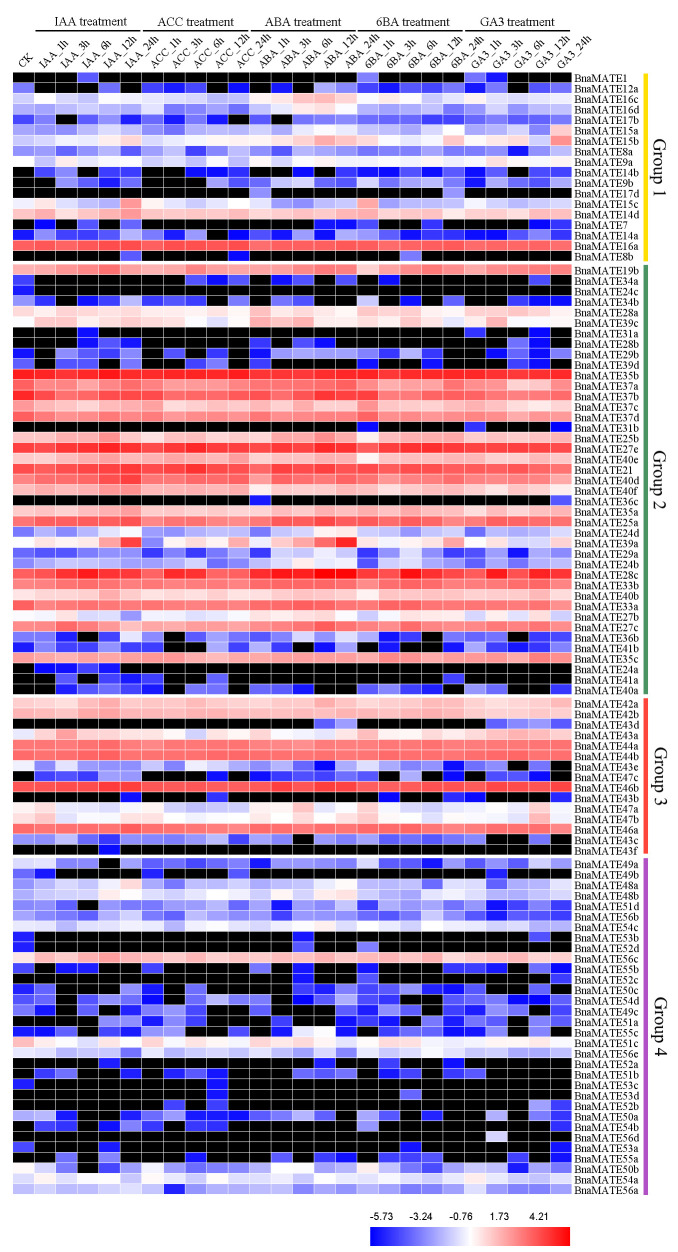
Expression analysis of *BnaMATE* family genes under hormone treatments. The color bar represents log_2_ expression levels (FPKM). Red, blue, and white indicate high, low, and medium expression levels, respectively, and black indicates no expression (FPKM = 0). 18 *BnaMATE* genes are not shown because their expression was not detected in any condition. The abbreviations above the heatmap indicate the different hormones used to treat rapeseed plants (listed in Table S8).

In *A. thaliana*, MATE family proteins have significant roles in the transport of hormones, including salicylic acid (SA) and abscisic acid (ABA) [11,16,17,18]. To explore the temporally and spatially specific expression patterns of the *BnaMATE* genes under hormone treatments, we used the transcriptome sequencing datasets to analysis their expression patterns over the course of treatment with the hormones indoleacetic acid (IAA), aminocyclopropane carboxylic acid (ACC), ABA, gibberellic acid (GA3), and 6-benzyladenine (6BA) (Figure 7). The Group 2 *MATE* genes showed relatively higher expression after the various hormone treatments than genes in the other three groups, whereas the Group 4 *MATE* genes were the most weakly expressed and in many cases showed no expression (Figure 7). Some *BnaMATEs* were up-regulated under multiple hormone treatments, including *BnaMATE15b*, *BnaMATE19b*, *BnaMATE28c*, *BnaMATE39a*, and *BnaMATE43a*, whereas others were up-regulated by specific hormone treatment: for example, *BnaMATE16c* and *BnaMATE16d* were highly expressed under ABA treatment, whereas *BnaMATE39a*, *BnaMATE48a*, and *BnaMATE48b* seemed more sensitive to IAA and ABA. Other *BnaMATE* genes were down-regulated by hormones treatment, such as *BnaMATE12a*, *BnaMATE37a*, *BnaMATE37b*, *BnaMATE37c*, *BnaMATE37d*, *BnaMATE50a*, *BnaMATE50b*, and *BnaMATE51c*. Overall, these results suggest that many *BnaMATE* genes may play important roles in processes related to hormones.

## 3. Discussion

Multidrug and toxic compound extrusion (MATE) is a present categorized multidrug efflux transporter family in almost all prokaryotes and eukaryotes, which is a large family of secondary active transporters in all kingdoms of life [4,22]. Recently, numerous studies of the MATE family have been carried out in plants, in species including rice [20,21], maize [22], tomato [4], upland cotton [23], soybean [24] and potato [38], but they have rarely been studied in the Brassicaceae. However, the Brassicaceae, given their complex history of genome duplication, are a particularly good model for investigating polyploidy in evolution, which is a major evolutionary process in eukaryotes [29,33]. Thus, the completion of Brassicaceae genome sequencing provides an excellent opportunity for the genome-wide characterization of the *MATE* family in the major *Brassica* plants, which include three diploid (*B. rapa*, *B. nigra* and *B. oleracea*) and two allotetraploid species (*B. juncea* and *B. napus*). Based on our results, we identified 124, 85, 81, 79, and 130 *MATE* genes in *B. napus*, *B. rapa*, *B. oleracea*, *B. nigra,* and *B. juncea*, which in all cases exceeds the 57 found in *A. thaliana* [13] and is consistent with the past genome duplications. In addition, the phylogenetic tree was supported by the intron–exon structures and motif compositions, as these showed considerable conservation within each group and greater differences between groups, particularly in the numbers and sizes of introns and exons (Figure 2, Table S2). The exonization of intronic sequences and pseudoexonization of exonic sequences play major roles in the divergence of gene structure, which is another likely cause for the functional diversification of *MATE* genes [43]. Introns are thought to alter gene activities, and the presence of introns in the genome is believed to impose a substantial burden on the host. Interestingly, the only intronless *MATE* genes were found in Group 4, whose members contains a maximum of only two introns, which is consistent with the patterns in cotton and maize [22,39]; this strongly supports the likelihood that the expansion of the *MATE* genes in Brassicaceae was also governed by intron loss and gain.

Gene duplication is a major driving force in gene family evolution and expansion [44]. In this study, 113 out of 124 *BnaMATE* genes (91.1%) were derived from WGD or segmental duplication, suggesting that such duplication events were the main contributors to the expansion of the *BnaMATE* genes. To establish strong evidence for homology, we assessed microsynteny within the Brassicaceae genomes, as well as syntenic genes between the Brassicaceae and *A. thaliana* genomes. There were 57 *MATE* genes in *A. thaliana*, which would be expected to have 171 *MATE* genes in *B. rapa* and *B. oleracea* given the whole-genome triplication (WGT) that occurred in *Brassica* species [33,41], and 342 *MATE* genes in *B. napus*, which was generated through hybridization of the progenitor species *B. rapa* and *B. oleracea* 7500–12,500 years ago [29]. In actuality, we found 85 *BraMATE*, 81 *BolMATE,* and 124 *BnaMATE* genes, indicating that duplicated genes were lost after WGT. Since the divergence of *A. thaliana* and *Brassica*, about 35% of genes in *Brassica* have been lost via deletion [45]. In total, we identified 29 collinear gene pairs between *A. thaliana* and *B. rapa*, but only 17 collinear gene pairs between *A. thaliana* and *B. oleracea*, although this may be due to assembly errors in the currently available *B. oleracea* genome information. *B. napus*, as an allopolyploid, was created through the hybridization of its diploid progenitors *B. oleracea* and *B. rapa.* The number of *MATE* genes (124) in *B. napus* was far less than the sum of the *MATE* genes in *B. oleracea* (81) and *B. rapa* (85), indicating that *MATE* gene loss that occurred during the evolution of *B. napus*. *B. juncea* is descended from a hybridization between *B. rapa* and *B. nigra*. Gene loss occurred among the MATEs of *B. juncea* as well, such that the number of *MATE* genes (130) in *B. juncea* is likewise much lower than the sum of those in *B. rapa* (85) and *B. nigra* (79). When we determined the *K*_a_ and *K*_s_ values, an important tool in investigating the type of selection pressure acting on protein-coding genes [46], we found that, interestingly, the *K*_a_/*K*_s_ ratio was <1 for all gene pairs of *A. thaliana* and *B. napus* or *B. napus* and *B. napus*, indicating that the *BnaMATE* genes have undergone large-scale purifying selection.

Our synteny analysis between *B. napus* and its diploid progenitors *B. oleracea* and *B. rapa* indicated that most *Brassica MATE* genes were located in syntenic regions, with 52 gene pairs shared between the A_na_ subgenome of *B. napus* and the A_ra_ genome of *B. rapa*, 44 gene pairs shared between the C_na_ subgenome of *B. napus* and the C_ol_ genome of *B. oleracea*, 29 gene pairs shared between the A_ju_ subgenome of *B. juncea* and the A_na_ genome of *B. napus*, and 42 gene pairs shared between the B_ju_ subgenome of *B. juncea* and B_ni_ genome of *B. nigra*. Nonetheless, some *MATE* genes in diploid progenitors were lost. The progenitor species had different numbers of chromosomes, and the A, B, and C subgenomes have undergone rearrangements, during which genes were presumably lost in the process of polyploidization.

According to our phylogenetic analysis, these *MATE* genes were classified into four groups, which is congruent with previous findings [1,23,24], suggesting similar evolutionary trajectories among these plants. In contrast, the previous studies reported that five MATE subfamilies were identified in tomato [4] and *C*. *sinensis* [25], six were identified in potato [36], and seven were identified in maize [22], indicating a functional diversification among plant species in the complex plant processes. In addition, phylogenetic analysis revealed that all of the *MATE* family genes from Brassicaceae are closely associated with *AtMATE* genes within the same groups (Figure 1) and exhibited similar exon–intron structures (Figure 4), indicating that members within the subfamily have highly conserved and might have similar functions in the same groups [47]. Group 1 contains 26 *BnaMATE* genes along with *AtMATE1* from *A. thaliana*, whose protein product mediates the efflux of plant-derived or exogenous toxic compounds from the cytoplasm, conveying resistance to Cd^2+^ [13], while other members of Group 1 were rarely reported. Group 2 contains 51 *BnaMATE* genes, along with *A. thaliana* genes whose products are reported to mediate the transport of alkaloids and phenolic compounds and influence multidrug resistance, such as *AtMATE19*, *AtMATE33*, *AtMATE35,* and *AtMATE41*. *AtMATE19*, also known as *ALF5* (*ABERRANT LATERAL ROOT FORMATION* 5), encodes a protein involved in multidrug resistance [48]. The protein product of *AtMATE35*, also known as *FFT* (*FLOWER FLAVONOID TRANSPORTER*), was considered to be a flavonoid transporter [49]; however, other evidence suggests that *AtMATE35*, as well as *AtMATE33*, functions as a vacuolar chloride channel involved in cell turgescence during stomatal movements, root hair elongation, and pollen germination [50]. *AtMATE41* is also known as *TT12* (*TRANSPARENT TESTA 12*), and previous studies raised the possibility that the functional of *TT12* is a flavonoid transporter in *Arabidopsis*, which is highly specific expressed in developing seeds [7,51,52]. Among the *B. napus MATE* genes in Group 2, *BnaMATE19* genes, because of their close relation with characterized transporters, seem to be the best candidates for conveying the protection of roots against inhibitory compounds; the *BnaMATE33* and *BnaMATE35* genes most likely encode proteins that function as chloride channels essential for turgor regulation, which may also be involved in the transport phenolic compounds, along with *BnaMATE41*. Group 3 contains 15 *BnaMATE* genes along with *AtMATE42*, *AtMATE43,* and *AtMATE47* from *A. thaliana* and *BolMATE42a* from *B. oleracea*. *AtMATE42* encodes the root citrate transporter, which, together with the root malate transporter *ALMT1*, is involved in the primary mechanism of aluminum (Al) tolerance [53]. *AtMATE43* (*FRD3*, *FERRIC REDUCTASE DEFECTIVE 3*) encodes a protein responsible for loading citrate, a chelator of Fe, into the root xylem for efficient Fe translocation [54,55]. The *AtMATE47* (*EDS5*, *ENHANCED DISEASE SUSCEPTIBILITY*) protein product participates in SA-dependent signaling for disease resistance [12,56]. *BolMATE42a*, also called *BolMATE* (KF031944), is the homolog of an *A. thaliana* gene encoding a protein that activates citrate efflux upon exposure to Al^3+^, leading to enhanced Al tolerance [15].

Overall, these findings strongly suggest that *BnaMATE42*, *BnaMATE43,* and *BnaMATE47* may be related to Fe and Al homeostasis. Additionally, Group 4 contains 32 *BnaMATE* genes along with *AtMATE48*, *AtMATE50,* and *AtMATE51* from *A. thaliana*. *AtMATE48* (*BCD1*, *BUSH, AND CHLOROTIC DWARF 1*) is involved in maintaining cellular Fe homeostasis by secreting excess Fe [57], whereas *AtMATE50* acts as an ABA efflux transporter [18]; *AtMATE51* (*ADS1*, *ACTIVATED DISEASE SUSCEPTIBILITY 1*) negatively regulates the accumulation of the plant immune activator SA for disease resistance [11]. Thus, members of this group, especially *BnaMATE48*, *BnaMATE50,* and *BnaMATE51*, encode proteins involved in ABA and SA efflux and Fe tolerance mechanisms.

Gene expression analysis is widely considered a valuable tool for obtaining fundamental information on the possible functions of genes under study [22,39]. To further analyze the temporally and spatially specific expression patterns of *BnaMATE* genes, we defined their transcription levels in 15 tissues at different developmental stages. We found that most showed differing expression in different tissues (Figure 5), implying that individual *MATE* genes may play specific roles in the development of individual tissues under normal conditions. The transcription levels of Group 2 *BnaMATE* genes were higher than those for the other three groups, indicating that the Group 2 genes may be important throughout *B. napus* development. It is well established that tissue-specific gene expression plays a crucial role in developmental and other physiological processes in specific tissue [58,59]. Among the *BnaMATE* genes, *BnaMATE19a* and *BnaMATE19c* seemed to have high expression levels late in development (Figure 5), and both were orthologous to *AtMATE19*, which is involved in multidrug resistance [48]. *BnaMATE33a*, *BnaMATE33b,* and *BnaMATE35c* were specifically expressed at high levels in floral organs (Figure 5), and they were orthologous to *AtDTX33* and *AtDTX35*, respectively, which are thought to function as vacuolar chloride channels involved in cell turgescence [50]; *AtMATE35* is also considered to be a flavonoid transporter [48]. *BnaMATE35b* and *BnaMATE35d* were highly expressed in floral organs, seed coat, and silique pods (Figure 5), suggesting that those proteins may have the same function as *AtMATE35*. *BnaMATE41a* and *BnaMATE41b* had high expression levels during the prometaphase of seed development (Figure 5), which is in accordance with previous studies [7], and they were orthologous to *AtMATE41* (*TT12*), implying that the proteins could play a similar role in flavonoid transport [7,50,51]. *BnaMATE37c* and *BnaMATE37d* were highly expressed in all of tissues tested, and *BnaMATE24* genes were strongly specifically expressed in seeds, embryos, and seed coat, but no information is available on the functions of these genes or their homologs. Interestingly, we found that *BnaMATE47a* and *BnaMATE47b* had a high expression in silique pods (at 30, 40, and 46 DAF) and were probably necessary for SA-dependent signaling, given their close phylogenetic relationship with *A. thaliana AtMATE47* [12,56]. We also found tissue-specific gene expression in Group 4 genes: *BnaMATE56a*, *BnaMATE56b*, *BnaMATE56c*, *BnaMATE56d,* and *BnaMATE56e* were preferentially expressed in seeds and seed coat. These *BnaMATE56* genes were orthologous to *AtMATE56*, which encodes a protein that participates in increasing CO_2_ and stomatal closure [60]. It seems that the function of the *BnaMATE56* genes has been diverted over evolutionary time. Overall, the results revealed that most duplicated *BnaMATE* gene pairs had diverse expression patterns, suggesting that the functions of duplicated genes have tended to diversify, conforming to a major theme of long-term evolution.

Having established these basic patterns of *MATE* gene expression, we next sought to assess how these are influenced by plant exposure to abiotic stress. Heavy metal (Cd^2+^ and As^3+^) pollution in agricultural soils is a critical problem affecting crop production and quality, and the expression of some *MATE* genes is reported to increase in response to various abiotic stresses in plants [13,14,15,61,62,63]. Therefore, we carried out transcriptome expression profiling in *B. napus* seeds under As^3+^ and Cd^2+^ stress, which showed expression changes in *BnaMATE* genes in response to As^3+^ and/or Cd^2+^ stress. For instance, *BnaMATE1* showed higher expression under Cd^2+^ treatment (Figure 6); this was consistent with its status as the homolog of *AtMATE1*, which is involved in Cd^2+^ detoxification [13], and it strongly suggests that *BnaMATE1* might play an important role in Cd^2+^ tolerance. *BnaMATE6a* and *BnaMATE6b* showed expression patterns similar to that of *BnaMATE1*, implying that they may have similar functions. Furthermore, 18 and 26 genes were observably up-regulated (to >2-fold) under As^3+^ or Cd^2+^ treatment, respectively, compared to control conditions (CK), and 25 and 19 genes were down-regulated (to <0.5-fold); thus, all of these genes may have specific roles in As^3+^ and Cd^2+^ stress response. We also noted that some pairs of duplicated genes showed a distinct divergence of expression levels: for example, *BnaMATE43b* was down-regulated under As^3+^ or Cd^2+^ treatment, whereas *BnaMATE43e* was up-regulated. These results implied that these duplicated genes have developed diverse functions in response to As^3+^ and Cd^2+^ stress in the evolutionary history.

Previous studies have confirmed that *MATE* genes act in hormone transport [16,17,18]. We analyzed the expression profile of the *BnaMATE* genes in plants treated with five separate hormones for different periods of time through transcriptome expression profiling (Figure 7). We found a variety of expression patterns among *BnaMATE* family genes. For instance, *BnaMATE27e*, *BnaMATE35c*, *BnaMATE42a*, *BnaMATE42b*, *BnaMATE44a*, *BnaMATE44b*, *BnaMATE46a*, *BnaMATE46b* were not induced in response to hormone treatments, whereas *BnaMATE12a*, *BnaMATE37a*, *BnaMATE37b*, *BnaMATE37c*, *BnaMATE37d*, *BnaMATE50a*, *BnaMATE50b*, and *BnaMATE51c* were down-regulated, and *BnaMATE15b*, *BnaMATE19b*, *BnaMATE39a*, *BnaMATE28c,* and *BnaMATE43a* were up-regulated. Interestingly, *BnaMATE16c* and *BnaMATE16d* were specifically up-regulated under ABA treatment, implying that they may function in stomatal closure [64,65], and *BnaMATE39a*, *BnaMATE48a,* and *BnaMATE48b* seemed more sensitive to IAA and ABA than to other hormones (Figure 7). Since *BnaMATE50a* and *BnaMATE50b* were orthologous to *AtMATE50*, which acts as an ABA efflux transporter [18], and *BnaMATE51c* was orthologous to *AtMATE51*, which negatively regulates the accumulation of the plant immune activator SA for disease resistance [11], we believe that these *BnaMATEs* also have specific roles in responses to different hormones. Our results showed the first comprehensive analysis of *MATE* family genes and investigated expression profiles of *BnaMATEs* under heavy metals or hormones treatment, laying the foundation for further elucidating the roles of *MATE* genes in the export of toxins and other substrates.

## 4. Materials and Methods

### 4.1. Identification of MATE Transporters in Brassicaceae Species

The MATE transporter gene family has at least 56 members in the *A. thaliana* genome [13], and a 57^th^ gene, *AT2G04066* (Gene ID: 814942), is described by the term “MATE efflux family protein” in the NCBI (National Center for Biotechnology Information) database [66] and was therefore included in this study. The protein sequences of the 57 MATE transporters were downloaded from The Arabidopsis Information Resource (TAIR) database (Table S2) [67]. Using 57 AtMATE sequences as queries, a BLASTp analysis [68] was performed against whole-genome sequences in the BRAD database [37]. The candidate sequences were chosen based on an *E*-value of ≤1 × 10^–30^ and further analyzed using Pfamscan (http://www.ebi.ac.uk/Tools/pfa/pfamscan/) to confirm the presence of a MATE domain (PF01554.18, MATE). BLAST analysis of the MATE protein sequences in the *B. napus* genome database was performed using Geneious 4.8.5 software (http://www.geneious.com/; Biomatters, Auckland, New Zealand). The candidate genes were named using two- or three-letter abbreviations (italicized) denoting the source organism, the family name, and the positions in the subtribe, e.g., *BnaMATE1a*. The *A. thaliana* sequences were named following the generic system proposed for *A. thaliana* as *AtMATE1* to *AtMATE57*, respectively [13].

Genomic, protein, and coding sequences from *A. thaliana* were downloaded from TAIR and those from *B. napus* (v5), *B. rapa* (v3.0), *B. oleracea* (v1.1), *B. juncea* (v1.5), and *B. nigra* (v1.1) were downloaded from the BRAD database. The physicochemical properties of the deduced proteins, including the molecular weight (MW) and isoelectric point (pI), were determined using the ExPASy-ProtParam tool (http://web.expasy.org/protparam/) [69].

### 4.2. Subcellular Location Analysis of Brassicaceae MATE Transporters

Subcellular location prediction of the candidate MATE proteins was performed with the online TargetP1.1 (http://www.cbs.dtu.dk/services/TargetP/) server [70] and Protein Prowler Subcellular Localization Predictor version 1.2 (http://bioinf.scmb.uq.edu.au/Pprowler_webapp_1-2/) [71]. Validation and determination of the possible cell compartmentalization, as suggested by the two software programs, was done with WoLF PSORT (https://wolfpsort.hgc.jp/) [72].

### 4.3. Phylogenetic Analysis of Brassicaceae MATE Transporters

The protein sequences of the MATE proteins from *A. thaliana* and *B. napus* were used for multiple protein sequence alignments selected with MUSCLE [73] by Molecular Evolutionary Genetics Analysis (MEGA) 7.0 software (Arizona State University, Tempe, Arizona, USA) [74]. Phylogenetic trees were built using the neighbor-joining (NJ) method with 1000 replicates for the bootstrap test [75], and the evolutionary distances were computed using the Poisson correction method [76]. The phylogenetic trees were visualized using FigTree v1.4.2 (http://tree.bio.ed.ac.uk/software/figtree/).

### 4.4. Chromosomal Distribution, Gene Duplication, and Evolutionary Analysis of Brassicaceae MATE Genes

All *MATE* genes were mapped to the chromosomes of Brassicaceae species according to their physical distances in the GFF genome files, which were downloaded from BRAD [37]. The physical chromosome maps were visualized with MapChart 2.2 (Plant Research International, Wageningen, Netherlands) [77]. Multiple collinear scanning toolkits (MCScanX) (University of Georgia, Athens, GA, USA) with the default parameters were used to view collinearity and analyze the gene replication events between *A. thaliana* and the five Brassicaceae species [78].

TBtools was used to construct a schematic diagram of the putative duplications of the *MATE* genes [79] and calculate the ratio of the nonsynonymous substitution rate (*K*_a_) to the synonymous substitution rate (*K*_s_) with the NG (Nei and Gojobori) method [80]. Divergence time was inferred using the formula *T* = *K*_s_/2*R*, where *R* is 1.5 × 10^−8^ synonymous substitutions per site per year [81].

### 4.5. Gene Structure and Conserved Motif Analysis of BnaMATE Genes

The Gene Structure Display Server (GSDS 2.0: http://gsds.cbi.pku.edu.cn/) [42] was used to analyze the coding regions and genomic DNA sequences of the candidate *MATE* genes from *A. thaliana* and *B. napus* to determine their exon–intron structures. The conserved domains of the MATE protein family were predicted by the NCBI conserved domain search (www.ncbi.nlm.nih.gov/Structure/cdd/wrpsb.cgi) and further confirmed by conducting a Pfam database search. Conserved motifs were determined using MEME 5.0.1 (http://meme-suite.org/) with the following parameters—Motif width, between 6 and 300 residues; Number of repetitions, any; maximum number of motifs, 12 [19] and visualized using TBtools (https://github.com/CJ-Chen/TBtools) [79].

### 4.6. Expression Patterns of BnaMATE Genes among Tissues and Developmental stages

To obtain a better understanding of the functions of the *BnaMATE* genes, we investigated their transcriptional levels in the different tissues and organs (roots, stems, leaves, anthocaulus, calyx, petal, pistils, stamens, anthers, filaments, the top of main inflorescence flowers, seeds, embryos, seed coat, and silique pods) of *B. napus* Zhongshuang No. 11 (ZS11) at different developmental stages. The resulting data were deposited in BioProject with ID PRJNA358784. The fragments per kilobase per million mapped fragments (FPKM) values were normalized using log_2_ transformation, and then the similarities and differences of the *BnaMATE* genes were visualized with heatmaps drafted by using Heatmap Illustrator 1.0 (HemI 1.0) (Huazhong University of Science and Technology, Wuhan, Hubei, China) [82].

### 4.7. Transcriptome Analysis of BnaMATE Genes in Response to Abiotic Stresses

The expression profiles of *BnaMATE* genes under heavy metal (As^3+^ and Cd^2+^) [83] and hormone (BioProject ID PRJNA608211) treatments were investigated by RNA sequencing (RNA-seq). The plant materials and heavy metal and hormone treatments were as described in previous work [83,84,85]. The expression profiles of candidate *BnaMATE* genes were calculated based on FPKM values and visualized as heatmaps by using HemI 1.0 [82].

## Figures and Tables

**Table 1:** Statistics for *MATE* genes in each subgroup between *A. thaliana* and five Brassicaceae species.

Type	Family	*A. thaliana*	*B. napus*	*B. oleracea*	*B. rapa*	*B. juncea*	*B. nigra*
Group 1	MATE1	*AtMATE1*	1	/	1	2	/
	MATE1	*AtMATE2*	/	/	/	/	/
	MATE1	*AtMATE3*	*/*	1	/	1	/
	MATE1	*AtMATE4*	/	1	1	/	1
	MATE1	*AtMATE5*	/	/	/	1	1
	MATE1	*AtMATE6*	2	/	1	1	/
	MATE1	*AtMATE7*	1	/	1	2	2
	MATE1	*AtMATE8*	2	3	1	2	1
	MATE1	*AtMATE9*	2	2	2	2	1
	MATE1	*AtMATE10*	/	/	/	/	/
	MATE1	*AtMATE11*	/	/	/	/	/
	MATE1	*AtMATE12*	2	1	1	/	/
	MATE1	*AtMATE13*	/	2	2	5	3
	MATE1	*AtMATE14*	4	5	4	7	5
	MATE1	*AtMATE15*	4	2	1	2	2
	MATE1	*AtMATE16*	4	2	3	3	2
	MATE1	*AtMATE17*	4	2	2	3	3
	MATE1	*AtMATE57*	/	/	/	/	/
Group 2	MATE2	*AtMATE18*	/	/	/	3	/
	MATE2	*AtMATE19*	3	2	2	2	5
	MATE2	*AtMATE20*	/	/	/	/	/
	MATE2	*AtMATE21*	1	1	2	2	1
	MATE2	*AtMATE22*	/	/	/	/	/
	MATE2	*AtMATE23*	/	/	1	1	1
	MATE2	*AtMATE24*	4	2	2	3	1
	MATE2	*AtMATE25*	2	2	2	3	2
	MATE2	*AtMATE26*	/	/	/	/	/
	MATE2	*AtMATE27*	5	5	4	2	4
	MATE2	*AtMATE28*	3	1	1	/	2
	MATE2	*AtMATE29*	3	1	1	2	1
	MATE2	*AtMATE30*	/	/	1	/	/
	MATE2	*AtMATE31*	2	/	1	2	2
	MATE2	*AtMATE32*	/	/	/	/	/
	MATE2	*AtMATE33*	2	1	1	1	1
	MATE2	*AtMATE34*	2	1	/	2	1
	MATE2	*AtMATE35*	4	3	3	5	3
	MATE2	*AtMATE36*	3	3	2	2	2
	MATE2	*AtMATE37*	4	2	2	2	3
	MATE2	*AtMATE38*	/	2	4	2	2
	MATE2	*AtMATE39*	5	3	2	4	4
	MATE2	*AtMATE40*	6	4	5	3	4
	MATE2	*AtMATE41*	2	1	1	3	1
Group 3	MATE3	*AtMATE42*	2	2	2	3	2
	MATE3	*AtMATE43*	6	1	3	8	3
	MATE3	*AtMATE44*	2	/	1	2	1
	MATE3	*AtMATE45*	/	1	1	/	/
	MATE3	*AtMATE46*	2	2	2	1	/
	MATE3	*AtMATE47*	3	1	1	1	/
Group 4	MATE4	*AtMATE48*	2	2	1	3	1
	MATE4	*AtMATE49*	3	2	2	4	2
	MATE4	*AtMATE50*	3	2	2	6	1
	MATE4	*AtMATE51*	4	3	3	6	/
	MATE4	*AtMATE52*	4	/	2	5	/
	MATE4	*AtMATE53*	4	2	2	4	2
	MATE4	*AtMATE54*	4	2	2	3	2
	MATE4	*AtMATE55*	3	2	2	5	2
	MATE4	*AtMATE56*	5	4	2	4	2

## Supplementary Materials

Supplementary Materials can be found at https://www.mdpi.com/2223-7747/9/9/1072/s1. Table S1. The tissues and organs from *B. napus* ZS11 used in this study; Table S2. List of the identified *MATE* genes in *B. naps* genome; Table S3. *MATE* collinear genes in *A. thaliana*, *B. napus*, *B. oleracea*, *B. rapa*, *B. juncea* and *B. nigra*; Table S4. Gene duplication pattern in *B. napus*, *B. oleracea*, *B. rapa*, *B. nigra* and *B. juncea*; Table S5. The *K*_a_/*K*_s_ values duplicaiton time for duplicated *MATE* gene pairs in *B. napus*; Table S6. Heat map of expression levels of *B. napus MATE* family genes at the indicated growth periods and tissues and organs; Table S7 Comparison expression analysis of *BnaMATE* family genes in rapeseed between the CK and As^3+^/Cd^2+^ treatments; Table S8. Comparison expression analysis of *BnaMATE* family genes in rapeseed between the CK and hormones treatment; Figure S1. The detailed information of motif logos are obtained from the MEME Suite website, Figure S2: The detailed information of conserved domains of MATE proteins between *B. napus* and *A. thaliana.*
