# Supplementary figures and images for: A Genome-Wide Survey of MATE Transporters in Brassicaceae and Unveiling Their Expression Profiles under Abiotic Stress in Rapeseed
Source: Plants (Basel). 2020 Aug 20;9(9):1072. doi: 10.3390/plants9091072 (PMC7569899; doi:10.3390/plants9091072)

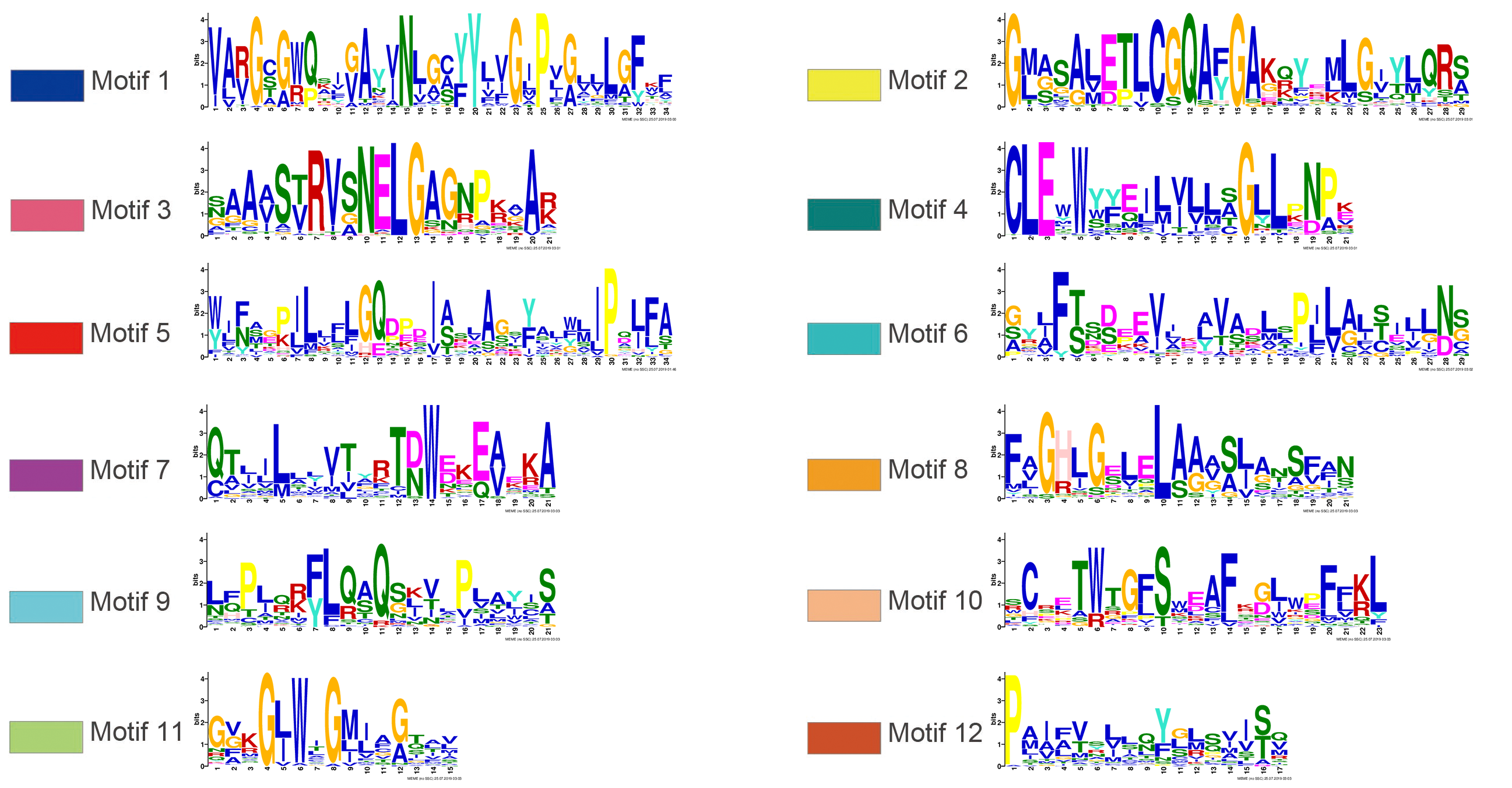

Supplement: Supplementary file 1 [file plants-09-01072-s001.zip › plants-885058-supplementary-new/Figure S1.tif]

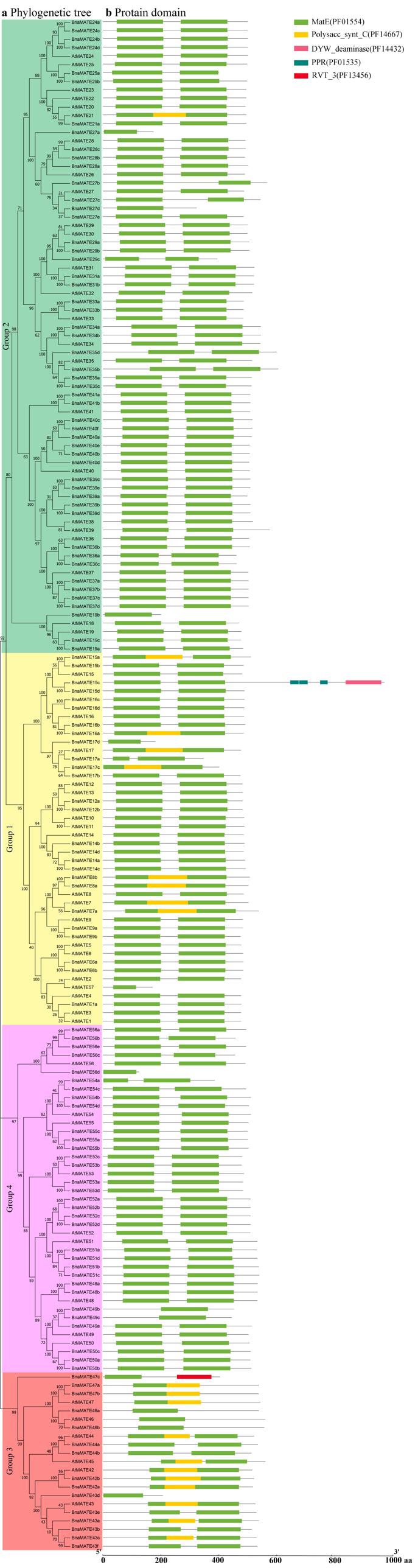

Supplement: Supplementary file 1 [file plants-09-01072-s001.zip › plants-885058-supplementary-new/Figure S2.tif]
